# Workflow and Practical Guidance for Identical Location Scanning Electron Microscopy: Reliable Tracking of Localized Transformations

**DOI:** 10.1002/smtd.202501290

**Published:** 2025-08-13

**Authors:** Blaž Tomc, Marjan Bele, Ana Rebeka Kamšek, Milena Martins, Aleš Marsel, Miha Hotko, Stefan Popović, Gregor Kapun, Črtomir Donik, Mitja Kostelec, Matjaž Godec, Nejc Hodnik, Luka Suhadolnik

**Affiliations:** ^1^ Department of Materials Chemistry National Institute of Chemistry Hajdrihova 19 Ljubljana 1000 Slovenia; ^2^ University of Nova Gorica Nova Gorica 5000 Slovenia; ^3^ Faculty of Chemistry and Chemical Technology University of Ljubljana Ljubljana 1000 Slovenia; ^4^ Department of Physics and Chemistry of Materials Institute of Metals and Technology Ljubljana 1000 Slovenia

**Keywords:** electrocatalysis, identical location electron microscopy, IL‐SEM, materials characterization, scanning electron microscopy, surface analysis

## Abstract

Understanding material transformations at the nano‐ and microscale is essential for advancing electrocatalysis, energy storage, and other applications. Conventional SEM imaging, which captures random locations before and after treatment, struggles to distinguish real transformations from inherent sample heterogeneity. Identical Location SEM (IL‐SEM) overcomes this by enabling re‐imaging of the exact same region, offering clear evidence of localized changes in morphology, structure, and composition. Despite its simplicity and wide applicability, IL‐SEM remains underutilized. This article presents a detailed, practical guide to implementing IL‐SEM reliably, including sample alignment, multiscale imaging, and consistent re‐localization. Key methodological tips and solutions to common challenges are provided, making the approach accessible even for non‐expert users. To showcase its versatility, we present case studies involving electrocatalysts, alloys, and nanostructured materials. Moreover, by integrating IL‐SEM with energy‐dispersive spectroscopy (EDS) and electron backscatter diffraction (EBSD), we demonstrate how compositional and crystallographic evolution can be tracked alongside morphological changes. This optimized workflow offers a powerful, non‐destructive method for visualizing dynamic material behavior and provides a foundation for IL‐SEM to become a standard technique for studying structural evolution across diverse materials research fields.

## Graphical abstract

Identical location SEM (IL‐SEM) is a powerful yet straightforward microscopy technique that enables precise tracking of material changes at the exact same location before and after different treatments. This article provides a practical, step‐by‐step workflow for successful IL‐SEM imaging and highlights the unique insights it offers across various research fields.

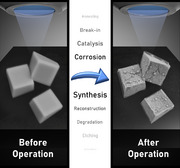

## 1 Introduction

The development of new materials is a cornerstone of technological progress, enabling advances in energy storage, conversion, and sustainability, as well as enhancing mechanical properties, corrosion resistance, and overall material performance. Catalysts, in particular, are critical enablers of chemical processes, allowing reactions to proceed efficiently, thereby supporting sustainable energy conversion or the green production of valuable chemicals. Designing and optimizing catalytic materials requires detailed insights into their (micro)structure, elemental composition, and behavior before, during, and after the operation,^[^ ^1^ ^]^ necessitating the use of advanced analytical methods. Such tools are essential for unraveling the complexities of material performance, especially in electrochemical applications where surface structure critically governs electrocatalytic activity.^[^ ^2^, ^3^, ^4^ ^]^ To capture these surface‐dependent phenomena, Scanning Electron Microscopy (SEM) offers a powerful and widely used platform for high‐resolution imaging of surface morphology and topographical features. Additionally, modern SEM systems integrate multiple detectors and analytical techniques that extend far beyond conventional imaging: i) Energy Dispersive X‐ray Spectroscopy (EDS) enables rapid elemental analysis by detecting characteristic X‐rays emitted during electron–sample interactions, ii) Electron Backscatter Diffraction (EBSD) provides crystallographic information, revealing grain orientation, phase distribution, and microstructural features such as grain boundaries, etc.^[^ ^5^, ^6^ ^]^

A key advantage of SEM is its ability to analyze materials non‐destructively, enabling repeated observations of the same specimen without compromising structural integrity. However, conventional SEM workflows typically involve randomly selecting areas for imaging before and after treatment, making it challenging to track localized and/or subtle changes over time. This limitation becomes especially critical in systems where performance is governed by dynamic, site‐specific transformations, such as in catalysis, energy storage, and surface modification.^[^ ^7^, ^8^ ^]^ To address this challenge and enable direct observation of material evolution at the exact same position, the concept of identical location SEM (IL‐SEM) was introduced in 2012. Meier et al.^[^ ^7^ ^]^ first demonstrated the approach using a transmission electron microscopy (TEM) grid and an SEM detector, while Hodnik et al.^[^ ^8^ ^]^ advanced the method by adapting it for use with a standard SEM sample holder. In contrast to identical location TEM (IL‐TEM), which was introduced earlier by Mayrhofer and Arenz in 2008^[^ ^9^ ^]^ and relies on thin samples supported on TEM grids, IL‐SEM can be applied directly to solid‐state specimens such as bulk metals and catalyst films. This flexibility allows for diverse treatments and analyses without compromising sample integrity, making IL‐SEM particularly powerful for studying structure–property relationships in surface science.

IL‐SEM protocol typically involves the following steps: i) the sample is prepared and marked with recognizable features to enable precise relocation, ii) initial SEM imaging is performed to establish a detailed morphological, structural, and compositional baseline of the region of interest, iii) the sample undergoes a treatment, such as electrochemical cycling, thermal exposure, or chemical modification, iv) post‐treatment SEM imaging is conducted under identical conditions, and v) the images are aligned and compared to extract quantitative information on structural or compositional changes over time. While conceptually straightforward, reliably relocating the identical region in the post‐treatment session remains a critical challenge, especially when surface features shift or degrade during the applied treatment.

Despite this limitation, numerous IL‐SEM studies have demonstrated that the method can be successfully applied across diverse sample types, treatments, and research protocols if a well‐defined and consistent workflow is followed. The method provided valuable insights across various applications. In electrocatalysis, IL‐SEM was primarily utilized to determine the (in)stability mechanisms of the material's surface during various reactions: i) CO_2_ reduction reaction (CO_2_RR),^[^ ^10^, ^11^, ^12^, ^13^, ^14^, ^15^, ^16^, ^17^, ^18^, ^19^ ^]^ ii) oxygen reduction reaction (ORR),^[^ ^7^, ^8^, ^20^, ^21^, ^22^, ^23^, ^24^, ^25^, ^26^, ^27^, ^28^, ^29^, ^30^ ^]^ iii) hydrogen evolution reaction (HER),^[^ ^31^, ^32^ ^]^ and iv) oxygen evolution reaction (OER).^[^ ^33^, ^34^, ^35^ ^]^ Beyond catalysis, IL‐SEM has been employed to monitor externally induced changes of various materials,^[^ ^36^, ^37^, ^38^, ^39^, ^40^, ^41^ ^]^ and it was also successfully utilized to capture distinct stages of material synthesis.^[^ ^42^, ^43^ ^]^ This underscores that the main barrier to broader adoption is not the execution itself, but the lack of accessible and practical guidance, which remains scarce or fragmented in the existing literature.

To address this gap, we present a comprehensive and experience‐driven IL‐SEM workflow that goes beyond previous descriptions by offering detailed, practice‐oriented instructions. Rather than merely compiling published information, this work distills years of hands‐on expertise to clarify methodological ambiguities and introduce a streamlined protocol supported by modern imaging tools. By systematically developing the method across diverse electrocatalytic systems and sample geometries, we gained unique insights that allowed us to establish a precise and broadly applicable IL‐SEM workflow. This process also revealed new application possibilities such as IL‐EDS and IL‐EBSD and helped define practical steps that, to the best of our knowledge, have not yet been documented in the literature.

While this study focuses primarily on electrocatalytic systems, the general principles, workflows, and analytical strategies are widely transferable. With its ability to precisely track nanoscale changes over time, IL‐SEM holds vast potential for broad applications in materials science, energy storage, corrosion studies, and even biological systems. By providing both conceptual clarity and practical guidance, we aim to promote IL‐SEM as a standard and scalable tool for tracking dynamic material transformations across diverse research fields.

## 2 Results and Discussion

### 2.1 IL‐SEM Concept and Detailed Workflow

As stated in the introduction, IL‐SEM enables the direct tracking of structural and compositional changes at the exact same location on a material's surface, offering valuable insights into dynamic processes. However, successful implementation depends critically on a clear and reproducible procedure for navigating back to the identical region after each treatment step. Unlike standard SEM analysis, the IL‐SEM method is specifically designed to repeatedly image and analyze the same area of a sample across multiple stages of synthesis, characterization, or treatment. This approach requires deliberate planning at every step to ensure successful relocation and consistent comparison.

**Figure 1:**
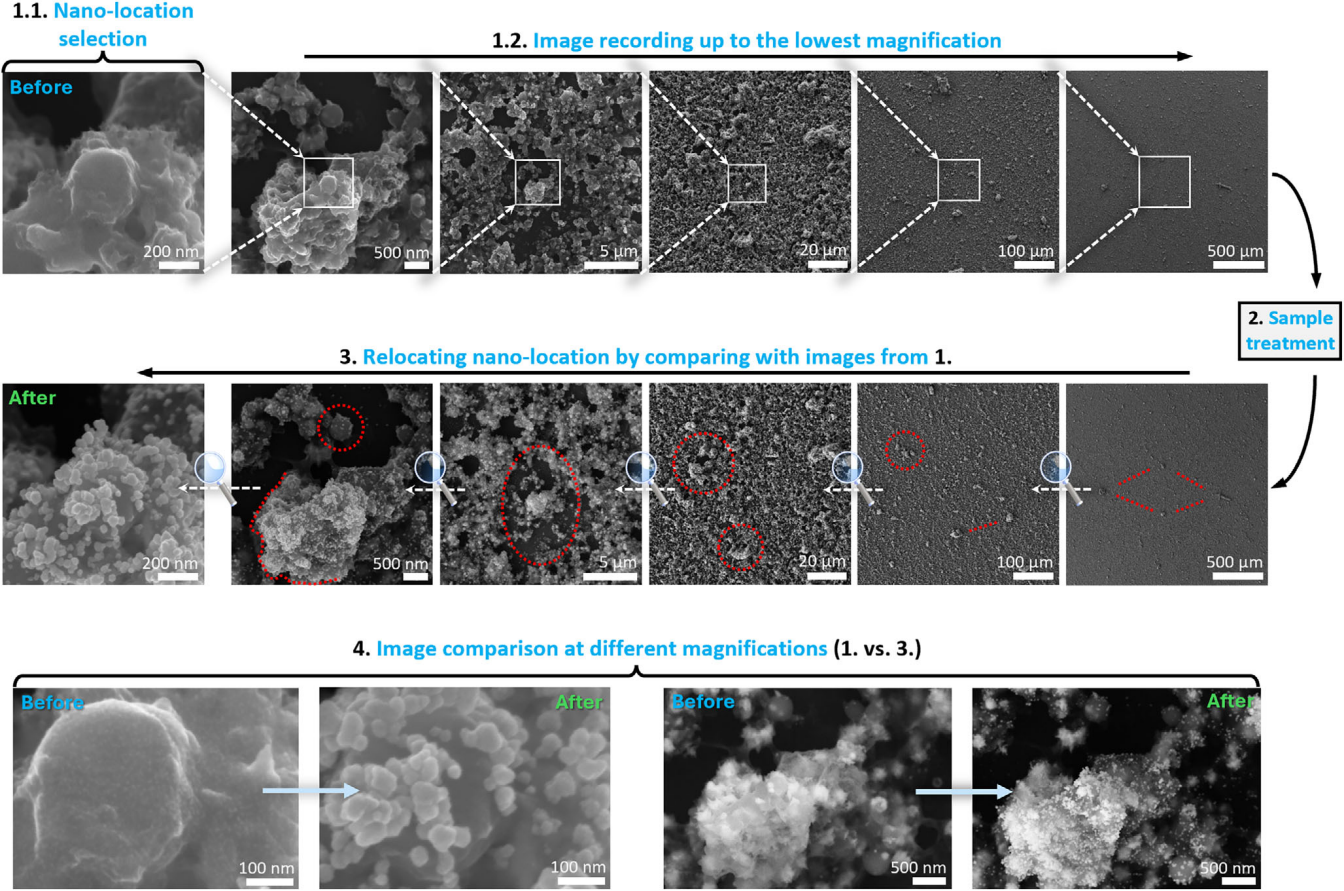
The typical IL‐SEM workflow consists of four steps, as described in the text. This case study illustrates copper nanostructured material embedded in carbon, analyzed before and after CO_2_RR. In step 1, a nano‐location was selected (1.1), and images were recorded at decreasing magnifications (200 000x–100x, 1.2). The sample was then retracted from the SEM in step 2, mounted in an electrochemical cell, and CO_2_RR was conducted at −0.93 V versus the reversible hydrogen electrode (RHE). Upon re‐insertion of the sample in step 3, the macro‐location was relocated by comparing images at 100x magnification, using a distinctive 4‐particle pattern (highlighted in red) as a reference. Gradually increasing the magnification enabled the precise identification of the nano‐location through distinct features. Finally, in step 4, comparing images at various magnifications revealed that the macro‐ and micro‐structures remained stable, while significant alterations were observed at the nanoscale, including the disappearance of sub‐5 nm copper nanoparticles and the formation of new Cu nanoparticles in the few–10 nm range. These findings underscore IL‐SEM's ability to capture material evolution, as further elaborated by Tomc et al., ^[^ ^11^ ^]^ from which a few SEM images in this figure have been adapted under a CC BY license for illustrative purposes.

The detailed IL‐SEM workflow typically consists of four straightforward steps, requiring no significant adjustments or advanced optimization (**Figure** **1**).

1. Sample insertion and imaging across scales: Insert the sample into the SEM and identify a nano‐location on the material where changes will be tracked (1.1). Then, capture SEM images starting from the highest down to the lowest magnification, moving from the nano‐ to the micro‐ to the macro‐scale (1.2).
2. Sample treatment: Retract the sample from the SEM, perform the desired treatment, and reinsert the sample into the SEM.
3. Relocating the identical location (IL) position: Use the recorded images from the first session as a guide to navigate from the macro‐ to micro‐ to nano‐scale and locate the identical position.
4. Image comparison: Capture new images at the IL position and compare them with the before‐treatment images to analyze changes.

In the following sections, we present a detailed IL‐SEM workflow and expand on each of the four key steps outlined above, providing practical guidance, methodological improvements, and insights gained through extensive experience. While some steps are not strictly required, they significantly enhance the reliability, efficiency, and success rate of the method, minimizing errors and accelerating the IL‐SEM discovery process. Based on our extensive experience over the past decade, the entire second SEM session (from sample reinsertion to image acquisition) can typically be completed in under 1 h, making the IL‐SEM method only marginally more time‐consuming than conventional SEM.

#### 2.1.1 IL‐SEM Analysis of the Initial Sample

**Figure 2:**
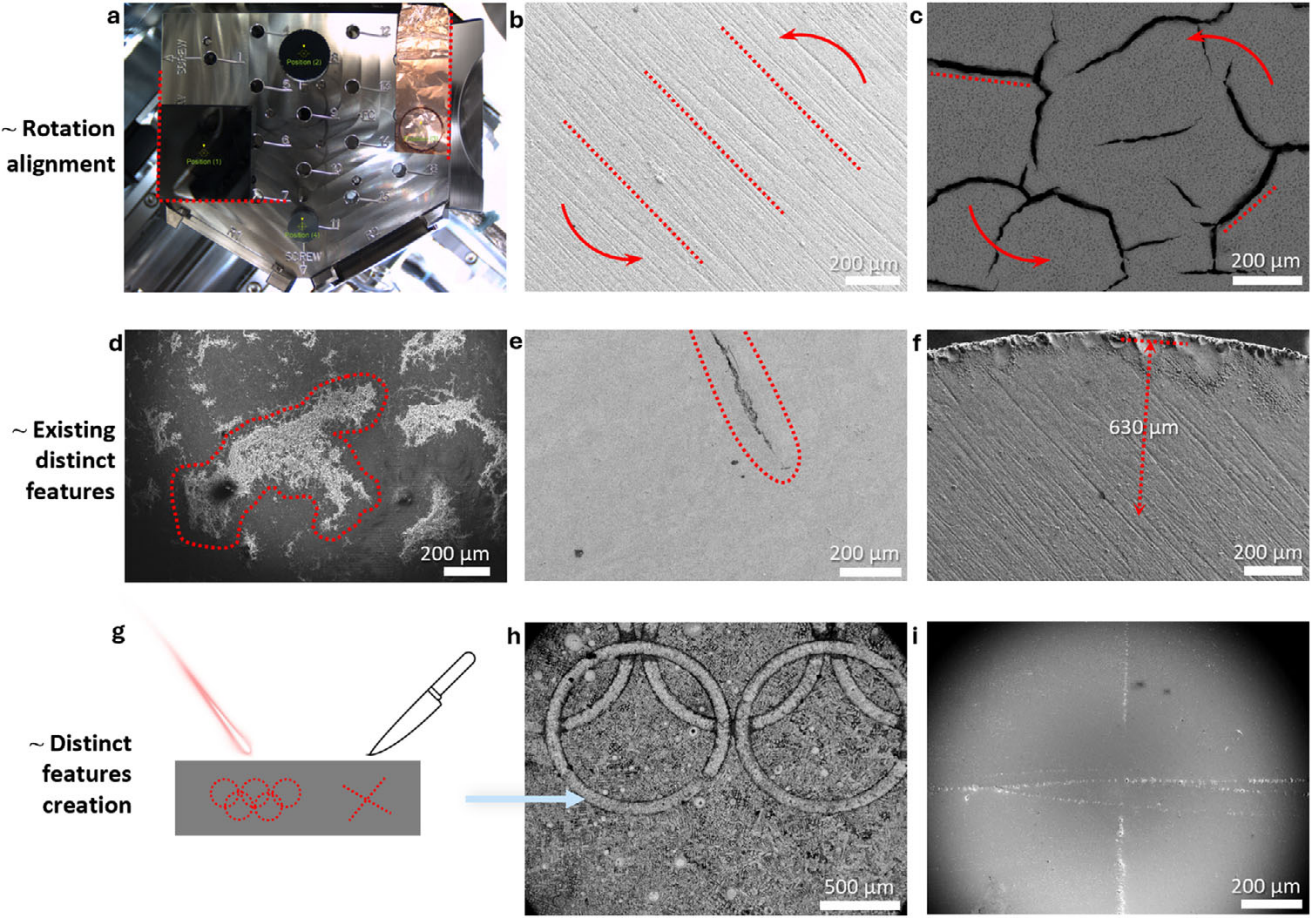
Different strategies for positioning the sample and selecting (creating) identifiable micro‐features during the initial IL‐SEM analysis to ensure the identical location can be easily relocated in subsequent sessions. These can include a) aligning the sample's edges with the stage's edges, selecting the middle of the sample as the location, saving the selected positions, and taking a Nav‐Cam photo, b,c) using perpendicular polishing lines (usually present on materials surface) or distinct material cracks (common for gas diffusion electrode; GDE) for rotation alignment if present, d–f) identifying distinct, naturally occurring material imperfections, typically present on all material types, as macro‐location markers to ensure easy relocation in subsequent sessions, f) measuring the distance between an edge imperfection and the desired location in samples without distinct features in the desired area of observation, and g–i) if no distinct macro features are present, e.g., single crystal studies and highly uniform films, artificial markers can be created by engraving, carving, or other methods to facilitate macro‐location repositioning.

Unlike conventional SEM analysis, which permits imaging of arbitrarily chosen areas, IL‐SEM requires strategic selection of one or more specific regions during the initial session. These regions must be carefully chosen based on identifiable features, orientation strategies, and expected changes, ensuring that they can be reliably relocated in subsequent analyses (**Figure** **2**). This process begins with macro‐level orientation and proceeds through multi‐scale imaging, selection of distinctive nano‐locations, and careful documentation using coordinates, images, and physical references.

The initial step involves orienting the sample, either during mounting on the SEM stage or by rotating the stage inside the microscope at low magnification, to achieve an orientation that can be easily recognized and reliably reproduced in future sessions (Figure 2a–c). If the sample has distinctive macro features observable by the naked eye, such as straight edges, corners, asymmetrical shapes, or labels and markings on the side or bottom, these can be aligned with the stage edges or mounted in a consistent orientation (Figure 2a). For instance, rotating the stage to a defined angle and setting the electron beam to 0°, aligning sample edges with stage features, and selecting the sample center as the IL region can allow even the micro‐location to be reliably recovered. A simple photograph of the mounted sample (e.g., using a mobile phone or Nav‐Cam, Figure 2a) further improves reproducibility, particularly in long‐term or collaborative studies. Additionally, if the SEM system allows, the macro‐location can be saved digitally, enabling automatic repositioning in future sessions if the sample is repositioned on the stage exactly.

In the absence of obvious geometric markers, surface micro‐features visible under SEM can be used for orientation. These include polishing lines (Figure 2b), cracks (Figure 2c,e), distinct shapes (Figure 2d), or small indentations near the sample edge (Figure 2f). These serve as reliable and treatment‐resistant reference points. Notably, indentations are preferred over protrusions, as the latter are more likely to degrade during sample handling or processing. When sample mounting does not permit reliable macro‐orientation, the stage can also be rotated at the micro‐location itself, provided the local features are distinct enough for unambiguous recognition.

If none of these features are available, manual creation of orientation markers is a simple and effective alternative (Figure 2g–i). Grazing, engraving, or scratching the surface or edge of the sample introduces distinctive, asymmetric features that serve as permanent reference points. These are especially useful for homogeneous or polished materials and can be positioned away from the IL region of interest. Importantly, these artificial markers compromise only a small fraction of the sample surface and do not influence the material's structural or functional properties.

Once the macro‐location has been identified, a photograph of the area is taken to document the position (Figure 2a). This image, along with notes on stage orientation and sample alignment, is saved in an electronic lab notebook to ensure that all team members, now or in the future, can reliably repeat the analysis.

When selecting multiple IL regions, only the primary IL location needs to be relocated using the standard macro‐to‐nano navigation approach. Secondary locations can be reached by recording and calculating relative stage coordinates based on the primary site. Our methodological advancement lies in the use of the SEM's internal navigation system to accurately record these coordinates and execute relative movements with high precision. This significantly increases the speed and efficiency of IL‐SEM, especially when characterizing heterogeneous samples where site‐specific variations (e.g., local morphology, composition, or bubble accumulation during electrochemistry) are expected. Additionally, choosing multiple locations provides redundancy, reducing the risk of losing critical data if one site becomes damaged or untraceable during treatment.

After selecting the general region of interest, the nano‐location is determined in the vicinity of the macro feature, and SEM images are captured across a range of magnifications, starting from the highest and progressively moving to lower magnifications (Figure 1). This multiscale imaging approach ensures that a wide range of spatial information is available to support accurate relocation and data comparison during future analyses. For example, typical magnifications used in our lab include 200 000x, 50 000x, 10 000x, 2000x, 500x, 100x, and the lowest magnification available on the microscope.

**Figure 3:**
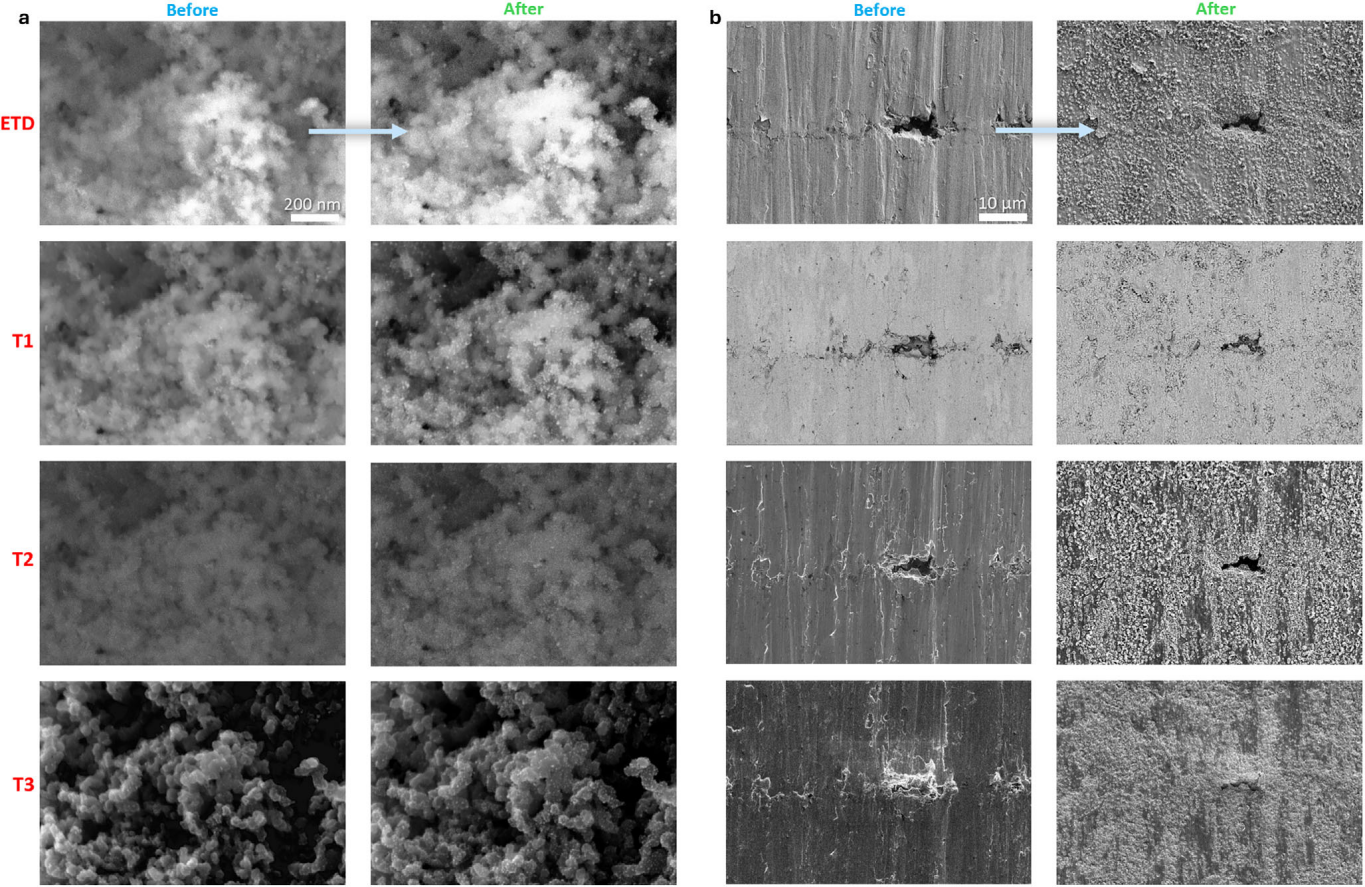
IL‐SEM images were acquired using four different detectors (ETD, T1, T2, T3) before and after two distinct treatments. a) Catalyst film of Pt nanoparticles supported on carbon and deposited on a GDE, imaged before and after electrochemical testing (10 000 successive 3‐s potential holds, alternating between 0.95 V and 0.55 V). b) Inconel 625 alloy surface, imaged before and after nitridation at 750 °C for 30 min in ammonia.

When the instrument permits, images are recorded using multiple detectors simultaneously to enhance data richness and provide complementary contrast mechanisms (**Figure** **3**). Each detector captures a different signal based on its design and position within the microscope, offering unique perspectives on surface or subsurface features. Some detectors are more sensitive to surface topography, while others are optimized for compositional or crystallographic contrast. Although these additional data are highly valuable, image comparison for IL tracking is typically performed using images acquired with the same detector, unless specific contrast differences are required.

For each magnification, the image providing the sharpest and most informative representation is selected. The choice of detector and operating parameters is critical, as these influence the type of signal collected and the depth from which it originates within the electron–sample interaction volume. For example, the conventional Everhart‐Thornley detector (ETD) captures secondary electrons (SE) from a broad region near the sample surface and is well suited for general topographic imaging. In contrast, in‐column detectors are optimized to detect high‐energy SEs emitted from a more confined interaction volume, enabling high‐resolution imaging of nanoscale features at high magnifications. Optimizing contrast and image quality is especially important for smooth or low‐contrast materials. Subtle features such as grain boundaries or surface defects can greatly assist in re‐identifying the IL position. Enhancing contrast during initial imaging can therefore improve the success rate of relocation in subsequent sessions.

This principle is demonstrated in Figure 3, which compares detector performance in two distinct systems. In Figure 3a, electrochemically cycled Pt nanoparticles supported on carbon become more visible due to particle growth. A combination of 4 detectors enables the choice of the most appropriate images for mechanistic interpretation. Since the ETD detector isn't sensitive to different atomic numbers (Z), the contrast provided is not sufficient for a reliable determination. On the other hand, T1 and T2 detectors, optimized for high Z sensitivity, reliably confirm the increased size and contrast of the Pt nanoparticles, with T1 providing the best signal and contrast. On the other hand, detector T3, which emphasizes surface topology, shows little change in the carbon support and depicts the changes of surface Pt nanoparticles. On the other hand, Figure 3b shows an Inconel 625 alloy surface after nitridation at 750 °C. The ETD and T3 detectors reveal pronounced surface roughening, while T1 and T2 confirm the formation of faceted precipitates consistent with step edges and defects reported in the literature.^[^ ^44^, ^45^ ^]^ These two examples corroborate the enhanced capabilities of imaging with several different detectors.

While SEM imaging provides essential morphological and structural information, even deeper insight can be gained by combining it with compositional analysis. In IL‐SEM, this is achieved through IL‐EDS analysis, which enables precise correlation between structural changes and elemental distributions over time. By performing EDS at the same locations imaged during SEM, one can directly link morphological evolution to compositional transformations, especially critical for the processes where materials are composed of several elements.

EDS analysis and EDS mapping are performed at the same location(s) identified during the SEM analysis. The appropriate magnification(s) for these analyses are carefully selected based on the goals of the study. For example, if the objective is to monitor small nanoparticles, a higher magnification is used to capture fine details and ensure accurate compositional mapping (Figure S2, Supporting Information). Conversely, if the goal is to analyze larger features or overall element distribution across a broader area, a lower magnification is more appropriate to provide a comprehensive overview (Figure S3, Supporting Information). The accelerating voltage and probe current are also crucial in EDS measurements, as lower voltages are used to capture more surface features, while higher accelerating voltages enable an analysis of layers a few micrometers beneath the surface, also depending on the material density. In contrast, the probe current is responsible for the number of electrons accelerated to the surface and the time necessary to perform the EDS mapping. When technology permits, it is also possible to conduct a detailed automated analysis over a larger region by dividing it into multiple smaller areas. The software then compiles these smaller datasets into a comprehensive, high‐resolution image. In such cases, the process, where the system methodically stitches together data from each analyzed segment, requires several hours and is typically run overnight to optimize time and ensure high‐quality results. This extended mapping approach is particularly valuable for examining the spatial distribution of elements in heterogeneous materials (Figure S3, Supporting Information).

When processing EDS mapping data, it is essential to consistently assign the same colors to identical elements across all mappings to simplify the interpretation of results and facilitate comparisons between different regions or stages of analysis (Figure S3, Supporting Information). However, during data analysis, it is also important to experiment with color settings and use the show/hide feature for individual elements. This approach helps to better understand the distribution of elements, as the combined color map can sometimes be misleading. For example, the overlay of two colors may create a third color that matches another element's assigned color, potentially confusing the relative changes in element alterations.

EDS analysis requires a dedicated analytical detector and cannot be performed simultaneously with ultra‐high resolution (UHR) SEM imaging at optimal conditions, particularly for IL‐SEM at identical nano‐locations. The main reason lies in the fact that EDS and SEM imaging rely on fundamentally different signals (X‐Rays versus SE) generated in distinct parts of the electron beam interaction volume and consequently demand different beam settings. For UHR SEM imaging, the use of low beam voltages and currents (resulting in a narrower, lower‐energy beam with fewer electrons) is essential for maximizing resolution by detecting the SE signal.^[^ ^5^ ^]^ These SEs originate from a significantly smaller volume very close to the sample surface, unlike X‐rays, which are generated deeper within the interaction volume. This difference allows SE imaging to achieve nanoscale lateral resolution, whereas EDS analysis is limited by its larger X‐ray analytical volume, which typically limits spatial resolution to a few hundred nanometers.^[^ ^5^ ^]^ It is also important to note that the working distance (WD) between sample and detector for X‐ray signal collection is defined by the solid angle of the EDS detector and is typically larger than the optimal WD for SE collection, which further impacts imaging precision, especially at the nanoscale. Therefore, SE imaging and EDS analyses are usually performed sequentially without removing the sample from the microscope to ensure optimal conditions for both imaging and elemental analysis.

Transitioning from SEM imaging to EDS analysis can, in certain conditions, cause a slight apparent shift in the sample's position, particularly at high magnifications and when working with specific imaging parameters. This shift is typically due to inserting the EDS detector or altering the beam current, both of which can influence the local electromagnetic field inside the microscope. However, at lower magnifications (below ∼10 000 ×) and when using consistent imaging parameters for both SEM and EDS, these effects are often negligible. For example, in metallurgical samples, where microstructural features such as inclusions, precipitates, and grain boundaries introduce natural heterogeneity, small shifts may not be as critical. Conversely, for nanoscale studies where precise location tracking is essential, such as in nanoparticle restructuring or catalyst evolution, any deviation can affect the direct comparison of SEM and EDS data. Therefore, repositioning to the IL location is essential when transitioning from SEM to EDS.

To ensure accurate alignment and streamline the transition from SEM imaging to EDS analysis, careful monitoring of the location is recommended. Performing a transition on a lower magnification and activating the center cross feature of the microscope can help track any movement and facilitate quick navigation back to the target area. Selecting a distinct, recognizable nano‐location further simplifies the realignment process, particularly in cases where high‐resolution compositional mapping is required. Regardless of the specific approach, performing EDS on the same region imaged with SEM ensures a more direct correlation between elemental and morphological data, enhancing the accuracy and depth of the analysis.

#### 2.1.2 Sample Treatment, Modification, and Additional Analyses

IL‐SEM becomes especially meaningful when the same region of a sample is imaged before and after a defined process, such as annealing, etching, electrochemical testing, etc., to assess how the treatment alters the material. These processes may be used to intentionally modify the material to enhance its performance or to evaluate its stability and behavior under operational conditions. A key requirement is that such treatments do not obscure or destroy the identifiable features needed to relocate the IL position. When combined with IL‐SEM, these interventions offer a powerful approach to directly correlate local structural and compositional changes with applied stress, enabling unique insights into the material's response and evolution.

In addition to the main treatment or testing step, complementary characterization techniques may also be applied to extract additional information about the material's surface, composition, or electronic structure.^[^ ^42^, ^43^ ^]^ However, care must be taken to avoid damaging the IL during these analyses, so that IL‐SEM imaging remains possible in subsequent sessions. For instance, deposition of carbon could occur during an IL‐EDS analysis (Figure S1, Supporting Information).

#### 2.1.3 Next IL‐SEM Analysis

Following the initial IL‐SEM session and any treatment, modification, or additional analysis, the second IL‐SEM session serves as a critical step to directly evaluate how these interventions have affected the material. Whether performed within the same day or after an extended period, the success of subsequent IL‐SEM analyses depends on how well the original session was documented. The aim is to faithfully reproduce the conditions and precisely relocate the identical nano‐location selected during the first session, allowing accurate tracking of local structural or compositional changes.

To begin, all materials from the initial IL‐SEM session should be readily accessible; this includes multiscale SEM images (Figure 1), photos documenting sample orientation and mounting (Figure 2a), notes on positional markers or distances (e.g., from sample edges), and any relevant metadata such as stage coordinates or microscope settings. These references streamline the navigation process and help contextualize observed changes, while also fostering productive discussion and hypothesis generation during the session. If a considerable time gap separates the analyses, proper sample storage becomes essential to preserve surface integrity. Samples should be stored and transferred in a vacuum‐sealed container (e.g., EM‐Storr boxes) using oil‐free pumps to minimize contamination from air, moisture, or particulates that could compromise surface‐sensitive imaging. The core objective of this session is to replicate the imaging conditions of the first IL‐SEM analysis as closely as possible. This includes restoring the same microscope parameters (e.g., accelerating voltage, WD, detector settings), achieving consistent sample orientation, and relocating the identical nano‐location with high precision.

To assist with realignment, several practical strategies can be used. Annotations on previous images, such as voltage, magnification, timestamps, or manual markers, serve as key reference points for matching frames and maintaining the correct orientation. Measuring distances from recognizable features, such as the sample edge, scratches, or intentionally introduced markers, helps approximate the macro‐position (Figure 2f). In addition, software tools like the center cross overlay available in many SEM interfaces can support fine‐tuning of the location during alignment; however, this feature should be turned off before final image acquisition to prevent the introduction of visual artifacts.

The re‐identification process begins with coarse positioning based on photographic documentation (Figure 2a). After loading the sample and configuring the microscope, navigation starts at the lowest magnification to identify macro‐scale features selected in the initial session (Figure 2c–f,h,i). If orientation was not fixed by stage alignment or visible patterns like polishing lines, the sample may require manual rotation at this stage to match the original configuration. Once the macro‐location is found, magnification is progressively increased while using stored reference images as a visual guide to home in on the nano‐location (Figure 1, step 3). Intermediate magnifications can often be skipped once the distinctive features of the IL site become apparent. Final alignment is done at the highest magnification to ensure optimal positioning. If available, the microscope's software can be used to save the precise stage coordinates for future access.

Once aligned, images are acquired in reverse order, from high to low magnification, using the same detector combinations and contrast settings as in the first session to ensure reliable comparison across sessions. Complementary analyses, such as quantitative EDS or EDS mapping, are carried out under the same conditions as in the initial analysis, maintaining consistency in beam settings and magnifications (Figures S2 and S3, Supporting Information).

After this analysis, the workflow returns to Step 2, where new treatments or measurements are applied, and Step 3 (IL‐SEM imaging) is repeated. This iterative loop enables continuous, site‐specific tracking of material evolution with high resolution and reliability. While the process may initially appear time‐consuming or complex, it becomes increasingly efficient with experience. For time‐intensive tasks such as high‐resolution EDS mapping over large areas, microscope use can be scheduled overnight to optimize lab time without compromising quality.

#### 2.1.4 Comparing Before and After IL‐SEM Data

**Figure 4:**
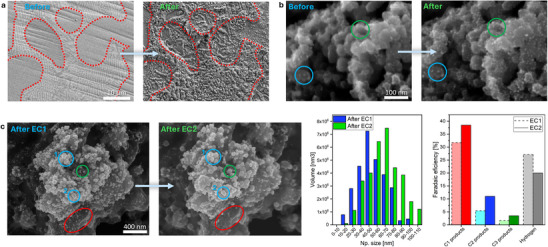
IL‐SEM examples. a) During annealing of the compositionally complex FeCoNiMoCu alloy, distinct restructuring behaviors are observed between the two phases, with alterations being significantly more pronounced in the stripped phase. b) This represents the first IL‐SEM analysis performed using a GDE setup with Pt nanoparticles on a carbon support, revealing important insights into the dynamic instability of Pt during electrochemical accelerated degradation test (10 000 successive 3‐s potential holds, alternating between 0.95 V and 0.55 V). Close inspection shows that the nanoparticles increase in size, most likely due to an Ostwald ripening‐type dissolution–redeposition mechanism. Interestingly, the extent of this mechanism appears to depend on the position and morphology of the catalyst film: the blue circle highlights a deeper region within the catalyst layer where the original nanoparticles persisted, while the green circle indicates a more exposed area where new nanoparticles formed or smaller ones, previously undetected, grew. c) Copper nanoparticles on carbon support restructuring during CO_2_RR at ‐0.895 V versus RHE through dissolution‐redeposition results in the formation of new and larger nanoparticles. EC1 and EC2 refer to the first and second electrochemical CO_2_ reduction experiments. The red ellipse represents the area where nanoparticles dissolved, two blue circles represent two particles that remained intact, while the green circle depicts a large copper nanoparticle that was newly formed. “After EC1” image was adapted from Tomc et al.^[^ ^11^ ^]^ under a CC BY license. d) Size distribution analysis (via histograms) revealed a clear shift in nanoparticle size toward larger values. Size distribution is presented as the volume of all nanoparticles in the distinct size region. Volume was calculated based on the nanoparticle radius with an approximation of a perfect sphere. e) The size shift of copper nanoparticles influenced CO_2_RR selectivity, resulting in decreased hydrogen production and increased hydrocarbon (C_1_, C_2_, and C_3_) formation.

**Figure 5:**
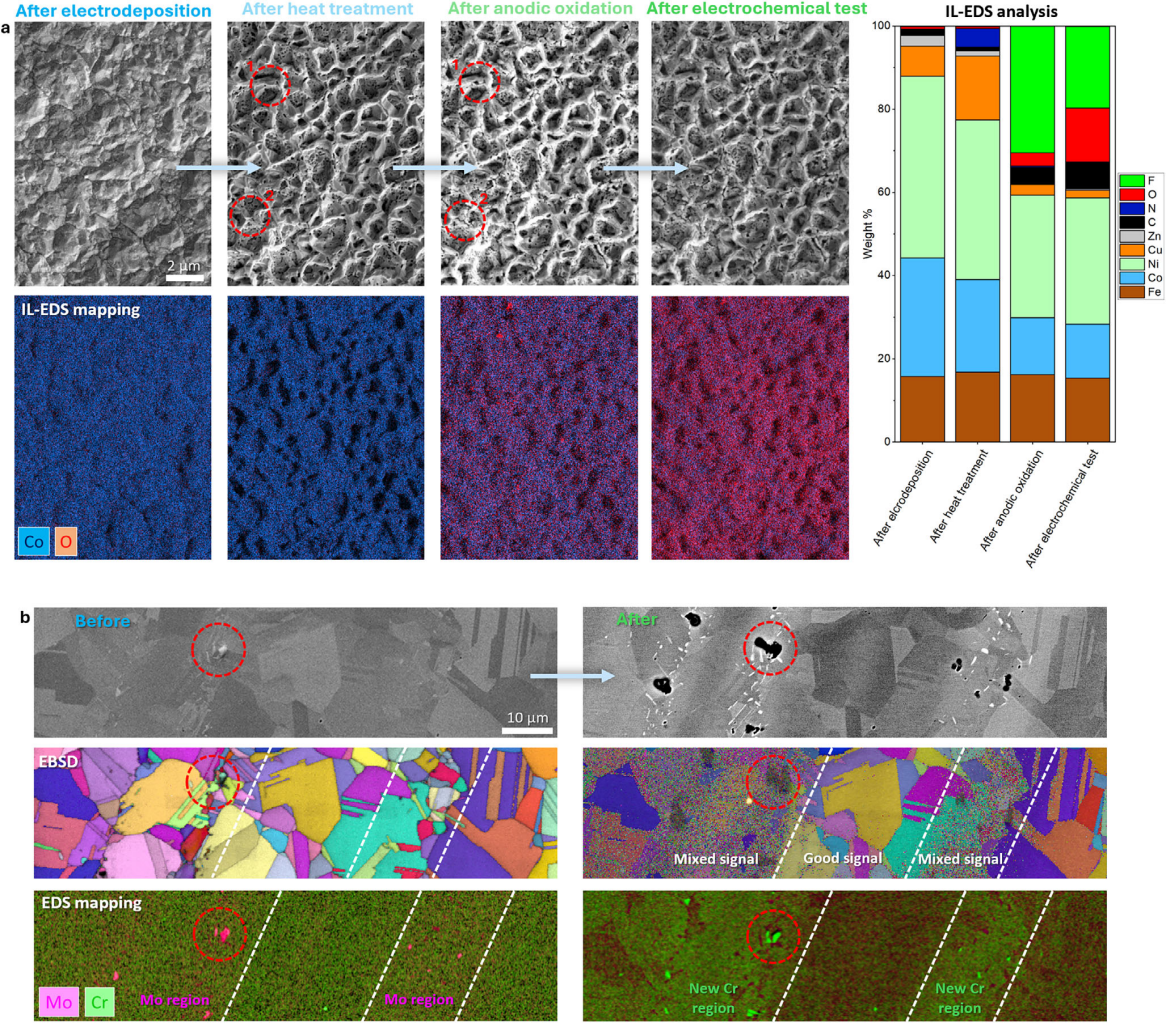
a) Electrodeposited FeCoNiCuZn high‐entropy alloy material was monitored across multiple stages of its cycle, from synthesis to electrochemical testing. Combined with IL‐EDS analysis and IL‐EDS mapping, this approach enabled precise tracking of structural and compositional changes. The red circles highlight barely visible cracks that formed during anodic oxidation. IL‐SEM‐EDS mapping of a larger area, showing individual element distributions, is presented in Figure S3, Supporting Information. b) IL‐SEM, IL‐EBSD, and IL‐EDS mapping analyses of the Ni‐based superalloy Inconel 625 before and after extended electrochemical cycling (50 cycles between 0.1 and 1.2 V vs. RHE, followed by 300 cycles in the 1.2–1.55 V vs. RHE range). The red circles indicate Mo‐rich regions before electrochemical cycling, which dissolved during the process, leaving behind voids. Additionally, Cr‐rich regions were identified, providing insights into the material's compositional evolution.

In IL‐SEM studies, the comparison of data collected before and after one or more treatments is critical for understanding material changes that result in performance changes. This comparison may involve analyzing both SEM images and EDS data to correlate morphological and compositional modifications across successive IL analyses. The before and after images should be placed next to each other and carefully examined, much like a scientific version of “spot the difference”.^[^ ^11^, ^46^ ^]^ Time and care must be taken to correctly evaluate all the changes. Utilizing the IL‐SEM approach, subtle but important alterations can be monitored (**Figures** **4** and **5**).

Example a: Phase‐specific evolution during material synthesis.

IL‐SEM proves invaluable in understanding material transformations during synthesis, as demonstrated in this example of heat treatment applied to a FeCoNiMoCu alloy consisting of two distinct phases (Figure 4a). The technique enables precise observation of changes occurring within each phase and at the phase boundaries—critical insights that would be impossible to obtain through random SEM imaging on a material of similar complexity.

In this experiment, IL‐SEM revealed how the two phases responded differently to heat treatment, with one phase exhibiting significantly more pronounced morphological changes than the other. Additionally, variations in behavior were observed within individual phases, offering further insights into the heterogeneity of the material's response. After nitridation in ammonia at 650 °C for 30 min, the dual‐phase region developed distinct surface crystallites, while the homogeneous region largely retained its smoother morphology. This indicates that the structural and possibly compositional differences between the two phases strongly influence how the material reacts to heat treatment. The observed crystallite formation in specific regions suggests localized diffusion or phase transformation mechanisms, which are critical to understanding functional properties in such complex alloys. Although further elemental analysis (e.g., EDS) would be needed to fully resolve the chemical processes involved, IL‐SEM alone already provides valuable spatial and morphological context. Such detailed observations, only achievable with IL‐SEM, provide a deeper understanding of phase‐specific transformations and the internal dynamics within each phase during synthesis. Furthermore, this approach minimizes interpretational bias by eliminating uncertainties related to imaging different locations across sessions, which is particularly beneficial in complex, multi‐phase materials. Crucially, the ability to precisely observe phase boundaries, where many of the most significant morphological and chemical changes occur, relies on direct comparison of identical locations and the careful tracking of characteristic features between sessions. Without this, key transformations at these interfaces could easily remain undetected or be misinterpreted.

Due to the substantial changes observed in the alloy after heat treatment, strictly adhering to IL‐SEM guidelines was essential to ensure the identical location could be easily found. Each session yielded detailed data, highlighting the challenge of comparing images from such complex systems. However, the IL‐SEM images provided crucial insights, enabling a precise understanding of the structural and compositional transformations resulting from the heat treatment process.

In this example, the IL was identified by first orienting the sample using an indentation on the sample edge, then measuring the distance toward the center to locate the approximate position (like Figure 2f). The final adjustment was made by recognizing characteristic phase patterns within the biphasic alloy, ensuring precise relocation in subsequent sessions (like Figure 2d). The high reproducibility of IL‐SEM across sessions was key to documenting the progression of structural changes with high spatial fidelity.

Example b: Tracking exact nanoparticle restructuring during electrochemical testing.

IL‐SEM was used to track the restructuring of sub‐10 nm Pt nanoparticles in a catalyst‐coated microporous layer on a gas diffusion layer (GDL) before and after 10 000 cycles of an accelerated stress test (AST), as proposed by the US Department of Energy (Figure 4b).^[^ ^47^ ^]^ It was previously revealed that Pt nanoparticles are unstable under these conditions, causing their dissolution due to oxide formation and reduction, resulting in the Ostwald ripening mechanism.^[^ ^47^, ^48^ ^]^ Monitoring identical locations revealed distinct nanoparticle transformations under conditions mimicking high‐current ORR fuel cell operation potentials (Figure 4b). The IL‐SEM analysis showed that Pt nanoparticles become more pronounced, indicating their growth via the Ostwald ripening mechanism. Interestingly, the position within the catalyst film also influences the extent of this mechanism (Figure 5b), with nanoparticles more exposed to the electrolyte exhibiting greater growth. This is consistent with previous observations in the rotating disk electrode setup and thus confirms the dynamic behavior of Pt^[^ ^7^, ^21^, ^23^ ^]^ even at an industry‐relevant setup, namely GDE. The non‐destructive nature of IL‐SEM further enables stepwise degradation studies on the same sample. Additional AST cycles could be performed to track progressive changes or intermediate IL‐SEM analyses could be conducted at defined intervals (e.g., after every 1000 cycles) to capture the evolution of degradation mechanisms in real time. In this example, the IL was identified by locating distinct cracks in the GDL, as shown in Figure 2c.

Example c: Tracking copper nanoparticle evolution during CO_2_RR.

Copper remains the only material capable of efficiently catalyzing CO_2_RR.^[^ ^49^ ^]^ However, despite its promise, the structure‐performance relationship in CO_2_RR is still not fully understood, largely due to continuous surface transformations driven by the dissolution‐redeposition mechanism during operation.^[^ ^10^, ^11^, ^50^, ^51^ ^]^ Among the various factors influencing performance, nanoparticle size plays a crucial role in dictating selectivity and efficiency.^[^ ^52^ ^]^ In this study, IL‐SEM provided direct insight into how copper nanoparticle size influences CO_2_RR selectivity. By analyzing the IL before and after sequential electrochemical treatments (EC1 and EC2), IL‐SEM revealed that smaller nanoparticles (∼0–40 nm) underwent dissolution, leading to the formation of larger ones (∼50–100 nm), while pre‐existing large nanoparticles (>50 nm) remained unaffected. The IL for this material was identified by locating distinct inhomogeneities, such as larger microstructures, as shown in Figure 1. This observation was possible through IL‐SEM, as conventional statistical SEM imaging cannot differentiate between changes caused by electrochemical transformation and those resulting from material exposure to the electrolyte.^[^ ^11^ ^]^ IL‐SEM enabled the identification of copper nanoparticles that remained entirely unaffected by both dissolution and redeposition. It also provided critical evidence that CO_2_RR suppresses copper electrodeposition,^[^ ^11^ ^]^ a conclusion that would be impossible to reach using conventional SEM techniques. Finally, it was possible to confidently correlate the enhancement of CO_2_RR selectivity, at the expense of suppressed HER, with the observed enlargement of copper nanoparticles, a trend that aligns well with findings by Reske et al.^[^ ^52^ ^]^

Example d: Morphological and compositional evolution during catalyst preparation and electrochemical testing.

IL‐SEM offers insights into how the morphology and composition (Figure 5a and Figure S3, Supporting Information) of FeCoNiCuZn high‐entropy alloy (HEA) electrodeposited onto a bulk metal substrate as OER catalyst evolve during catalyst preparation and testing. The process involved electrodeposition, heat treatment in ammonia, anodic oxidation in a fluorine‐containing electrolyte, and subsequent electrochemical testing. In this study, IL‐SEM imaging, IL‐EDS analysis, and IL‐EDS mapping were performed at the same location, allowing precise tracking of compositional changes throughout the process. After electrodeposition, the FeCoNiCuZn alloy exhibited a uniform surface with visible polishing lines serving as orientation markers, while EDS mapping confirmed the even distribution of elements across the electrodeposited film. Heat treatment induced step‐like morphological features and a strong decrease in Zn content, indicative of Zn sublimation. Anodic oxidation caused compositional shifts, including Cu dissolution from approximately 12 wt% to less than 3 wt% while F and O content significantly increased. These compositional shifts were accompanied by increased porosity. The final electrochemical test under OER‐relevant conditions resulted in further surface oxidation but without major morphological changes, indicating that a stable, high‐surface‐area structure had already formed during prior steps. This example clearly demonstrates how IL‐SEM enables the detection of even subtle structural and compositional changes that would otherwise go unnoticed. Without identical location tracking, one might mistakenly conclude that no further transformations occurred after heat treatment, as the surface appears largely unchanged at first glance. The ability to resolve such fine differences is essential for a true understanding of material evolution and catalyst behavior.

In this example, the IL was identified by aligning the sample via an edge indentation, measuring the distance toward the center (Figure 2f), and refining the position using elevated regions formed by faster electrodeposition. Identical locations are easy to find due to clear markings, thorough documentation, and the characteristic features formed during electrodeposition, which appear remarkably similar.

Example e: IL‐SEM‐EBSD‐EDS unveils molybdenum dissolution and surface evolution in Inconel 625 alloy.

IL‐SEM, IL‐EDS, and IL‐EBSD analyses were employed to investigate the microstructural and compositional evolution of Inconel 625 before and after extended electrochemical cycling (Figure 5c). Inconel 625, a high‐strength Ni‐Cr‐Mo alloy widely used in aerospace, nuclear, and chemical industries, was evaluated as an anode material for the OER due to its previously demonstrated stability in alkaline environments.^[^ ^53^ ^]^ The material underwent two electrochemical cycling protocols: 50 cycles between 0.1 and 1.2 V versus RHE, followed by 300 cycles in the 1.2–1.55 V versus RHE range.

Correlative IL‐SEM imaging and IL‐EBSD mapping enabled grain‐specific assessment of surface transformations at identical locations, with IL‐EBSD providing direct determination of grain orientations and sizes. Close examination of IL‐EBSD maps revealed a pronounced increase in noise and loss of pattern quality in regions composed of smaller grains and in areas containing defects such as Mo carbides after cycling (red dashed circles in Figure 5c). This degradation is attributed to the formation of a Ni‐based (oxy)hydroxide passive film, which can block EBSD signal acquisition even at thicknesses of a few tens of nanometers. The higher grain boundary density in fine‐grained regions accelerates localized corrosion and promotes thicker film growth. In contrast, larger grain areas developed thinner and more uniform passive layers, preserving clearer EBSD patterns. Grain boundaries themselves appeared particularly prone to intergranular corrosion,^[^ ^54^ ^]^ evidenced by highly degraded or absent EBSD signals along these features. This localized attack is likely driven by Cr depletion at grain boundaries, a phenomenon commonly observed in thermally treated Inconel alloys.^[^ ^55^ ^]^ The dissolution of Ni and other species at these sites likely led to the re‐precipitation and formation of a known OER‐active Ni (oxy)hydroxide‐rich film, further contributing to EBSD signal loss. These findings are consistent with literature reports on the stability of alloys, where grain‐size‐dependent corrosion rates are most pronounced in smaller grains due to their higher grain boundary‐to‐surface area ratio.^[^ ^56^ ^]^

In addition, IL‐SEM imaging revealed selective dissolution of Mo‐rich regions during cycling, leading to the formation of distinct voids (Figure 5b; red dashed circle). This observation was corroborated by IL‐EDS mapping, which confirmed complete Mo depletion at these sites. Concurrent IL‐EBSD analysis showed structural degradation, with diminished or lost Kikuchi patterns due to surface roughening and film formation. IL‐EDS further indicated increased Cr content in these regions, suggesting that Cr dissolution and re‐deposition occurred during anodic cycling, likely contributing to the preferential formation of Cr‐enriched Ni (oxy)hydroxide layers.

In this example, the IL was identified by aligning the sample via an edge indentation, measuring the distance toward the center (Figure 2f), and refining the position based on contrast variations from differently oriented grains, twin boundaries, grain shape, and other material imperfections such as segregations.

### 2.2 Advantages, Challenges, and Limitations of IL‐SEM

IL‐SEM provides a unique ability to analyze the exact same location on a sample across multiple stages of synthesis, treatment, or testing. This approach enables researchers to precisely identify and document changes in structure, composition, or morphology that occur as a result of a specific process. Unlike conventional SEM, where different locations might be analyzed before and after treatment, IL‐SEM ensures that observed differences are solely due to the applied process, eliminating ambiguity caused by natural heterogeneity in the material. However, as highlighted by Hou et al., careful consideration is needed during SEM analysis to avoid beam‐induced contamination, which can affect subsequent analyses and alter conclusions.^[^ ^57^ ^]^

A key advantage of IL‐SEM is that it removes the inherent subjectivity of random microscopy, where researchers may unintentionally or deliberately select regions that support a desired conclusion. In conventional SEM analysis, it is possible to search across a sample until the most convincing example is found—making it difficult to ensure whether observed differences genuinely result from a treatment or are simply a consequence of sample variation. This challenge has long contributed to skepticism toward microscopy‐based studies. IL‐SEM eliminates this uncertainty by forcing an objective comparison—the exact same region is analyzed before and after, ensuring that any observed changes are truly induced by the applied process rather than an artifact of sample heterogeneity.

By analyzing identical locations, researchers can confidently demonstrate and visually illustrate what has changed—or what has remained unchanged—during a specific process. This level of reliability is invaluable for understanding how materials behave under specific conditions, particularly when studying localized phenomena such as crack formation, surface etching, or compositional segregation (Figure 4d). Without the ability to analyze the same location, critical insights into material transformations can be lost, leading to incomplete or inaccurate conclusions.

IL‐SEM has several key advantages, even compared to operando techniques, providing precise tracking of structural and compositional changes without complex setups.^[^ ^58^, ^59^, ^60^ ^]^ Operando microscopy may also unintentionally alter materials through radiolysis,^[^ ^60^, ^61^, ^62^, ^63^ ^]^ potentially affecting their performance compared to experiments conducted without operando techniques. In comparison, more readily available IL‐SEM techniques retain a significant advantage over operando microscopy, particularly in terms of accessibility, ease of implementation, and maintaining material integrity during analysis.

Although IL transmission electron microscopy (IL‐TEM) can provide precise atomic‐scale observations of restructuring before and after treatments,^[^ ^27^ ^]^ its limited sample size, shape requirements, and sample loading impose several constraints. Additionally, the technique is less accessible due to the specialized equipment and expertise required. Moreover, recent advancements in SEM technology have significantly improved resolution while simultaneously reducing costs, making it a more viable option for a wider range of applications. Therefore, the use of IL‐SEM, which accommodates a wide variety of sample types, offers a significant advantage for widespread application in fields where samples are in a bulk solid state, such as materials and surface science, catalysis, and battery research. Unlike IL‐TEM, where a bulk sample must first be cut into thin lamellae—typically using focused ion beam (FIB) SEM—and then deposited onto a TEM grid, strictly defining the sample's size, thickness (preferably below ∼50 nm), shape, and compatibility, IL‐SEM allows for the analysis of a much broader range of samples (e.g., thick bulk metals or catalyst films), enabling their use in diverse treatments and analyses. This flexibility makes it possible to conduct experiments that are not feasible with IL‐TEM, further enhancing IL‐SEM's utility across multiple disciplines.

The analysis of IL significantly improves the depth of understanding by enabling direct comparisons. This approach is particularly beneficial in studying processes that induce subtle changes (Figure 4) where differences might be undetectable without exact spatial correlation. Furthermore, IL‐SEM incorporates complementary analyses, such as EDS and EBSD, within the same microscope setup (Figure 5, and Figures S2 and S3, Supporting Information). These additional capabilities enhance the understanding of material behavior by directly linking morphological changes observed through IL‐SEM with compositional or crystallographic data. This integrated approach allows researchers to build a more comprehensive and detailed picture of material transformations.

Although IL‐SEM offers numerous advantages, its implementation poses several challenges and limitations:

1. Locating the identical region: Successfully finding the same location requires precise documentation and a systematic approach. This becomes particularly challenging when significant time elapses between analyses, often due to the need for complementary analyses or multi‐step processes.^[^ ^43^ ^]^ Properly annotated images, detailed notes, and reference features on the sample are essential for ensuring reproducibility.
2. Avoiding sample damage: Throughout the entire process, it is critical to avoid damaging the IL to ensure accurate and consistent results. This is especially important in IL‐SEM studies, where typically only a single sample is available for analysis. If the sample is damaged or the IL is compromised, the entire study may need to be repeated—a process that can be extremely time‐consuming, particularly if numerous synthesis steps and analyses have already been performed. Strategic planning is essential to prevent unintended modifications to the key area of interest and maintain the integrity of the IL‐SEM location.
3. Equipment requirements: Although IL‐SEM can be performed with any SEM, having advanced features such as stage position memory, precise motorized stage controls, and several detectors working simultaneously significantly facilitates the process. Specialized microscopes or accessories, such as high‐resolution detectors or automated alignment tools, can further enhance the ease and accuracy of IL‐SEM.
4. Operator expertise: IL‐SEM requires skilled operators who are well‐versed in both the technique and the specific material being studied. Ideally, multiple operators work together, as collaboration can greatly facilitate the process of locating the identical region and ensuring all steps are followed correctly (Figure S4, Supporting Information). This teamwork helps maintain consistency, enables faster identification of the location, and ensures critical details—such as removing the center cross before capturing final images—are not overlooked. Having more than one operator also allows for cross‐checking and reduces the likelihood of errors, particularly during complex analyses. Additional tools like a large screen (Figure S4, Supporting Information) and a laser pointer can aid the process by enhancing visibility and precision.
5. Sample movement and preparation: During IL‐SEM analysis, the sample may shift slightly when conditions such as voltage or current are adjusted or when hardware, like the EDS detector, is inserted. These changes can cause the position of the sample to appear misaligned, requiring careful realignment to ensure the identical location is correctly identified.
6. Sample preparation and stability: Proper preparation and handling of the sample are crucial for successful IL‐SEM analysis. Cleanliness is equally important—the selected location must be free of debris or contamination that could obscure critical details. Steps such as rinsing the sample, blowing off particles, and storing it under clean, controlled conditions help preserve the integrity of the location and ensure high‐quality results. Neglecting these precautions can compromise the reliability of the analysis and obscure meaningful insights.
7. Time and effort: The meticulous nature of IL‐SEM, particularly for lengthy analyses such as large area EDS mapping, can be time‐consuming. Proper planning, such as scheduling overnight runs for time‐intensive processes, is crucial to optimize microscope usage and minimize interruptions. In addition, the process can be demanding for the operator, especially when the selected location is not immediately identifiable. This challenge can be alleviated by ensuring that the operator is not working alone. Having additional team members to assist with locating the region and cross‐referencing notes and images not only reduces the workload but also increases efficiency and accuracy during the analysis (Figure S4, Supporting Information). Collaboration makes the process smoother and helps maintain focus on the meticulous steps required for successful IL‐SEM.

The efficiency of IL‐SEM can be significantly enhanced with proper planning, collaboration, and advanced equipment. For instance, using the ThermoFisher Apreo 2S microscope, two operators can complete the imaging of a sample at 10 magnifications with four detectors at the identical location, perform an EDS analysis at 1 magnification, and prepare for an extended automated EDS mapping analysis—all within just 40 min. The EDS mapping process can then run overnight, ensuring optimal utilization of the microscope while minimizing downtime.

Although IL‐SEM introduces challenges in terms of equipment, expertise, and time, its ability to offer precise, reproducible insights into material behavior makes it an invaluable tool in materials science. By ensuring that ILs are analyzed, researchers can confidently attribute observed changes to specific processes, gaining a deeper and more accurate understanding of material transformations. Addressing the challenges through proper documentation, sample handling protocols, skilled operation, and advanced instrumentation ensures that IL‐SEM can be effectively integrated into complex research workflows.

### 2.3 Perspectives and Future Directions

A Scopus search (“scanning AND electron AND (microscopy OR microscope) AND NOT transmission” within article titles, abstracts, and keywords, excluding review papers) revealed over 631657 SEM studies in the past 25 years, highlighting the methods’ widespread use across disciplines. On the other hand, a similar search for IL‐SEM (“identical location” AND scanning AND electron AND (microscopy OR microscope) AND NOT transmission″) returned only 27 papers, indicating how rarely this method is used. Building on this foundational utility, IL‐SEM presents a transformative opportunity to deepen insights across disciplines by enabling precise, localized comparisons of IL on a sample. While this technique has already demonstrated significant contributions in materials synthesis and catalysis, its potential extends far beyond these fields, offering exciting possibilities for broader adoption.

In materials synthesis, IL‐SEM could be integrated into studies focusing on microstructure evolution,^[^ ^64^ ^]^ phase transformations (Figure 4a), surface modifications,^[^ ^65^ ^]^ and nanoparticle synthesis,^[^ ^66^ ^]^ providing unparalleled clarity in understanding how materials evolve during processing. Moreover, IL‐SEM holds immense value for other material research areas. Importantly, in corrosion studies,^[^ ^67^ ^]^ it could offer direct and detailed comparisons of the same location before and after treatment, enabling enhanced understanding of corrosion mechanisms. Similarly, in fatigue and fracture mechanics,^[^ ^68^ ^]^ IL‐SEM would enable researchers to track damage evolution at the exact same location, providing insights into material behavior during cyclic loading or fracture events—critical for industrial applications where reliability and performance are paramount.

Beyond traditional materials science, the versatility of IL‐SEM would be a powerful tool for a range of disciplines. In environmental science, it could elucidate material degradation in pollution control systems,^[^ ^69^ ^]^ or track microplastic breakdown,^[^ ^70^ ^]^ paving the way for more effective remediation strategies. In forensic science, IL‐SEM could enhance the analysis of trace evidence.^[^ ^71^ ^]^ Within energy storage research, IL‐SEM could provide detailed insights into electrode behavior, such as crack formation and surface transformations during charge‐discharge cycles,^[^ ^72^, ^73^ ^]^ advancing understanding of battery degradation mechanisms and informing the development of more robust energy storage devices. Likewise, the electronics industry could use IL‐SEM to investigate defect formation and wear in semiconductor devices,^[^ ^74^ ^]^ while food science applications might include monitoring structural changes in biodegradable packaging or food matrices under storage conditions.^[^ ^75^ ^]^

Therefore, the development and adoption of IL‐SEM holds immense potential for advancing not only materials science but also a wide range of fields. However, its impact and accessibility could be significantly enhanced through targeted improvements and innovations that make the technique more efficient, versatile, and user‐friendly.

A key area for advancement lies in the automation of both locating identical positions and analyzing the captured images.^[^ ^76^ ^]^ Integrating software capable of recognizing and aligning features across sessions would reduce reliance on manual navigation and annotation, streamlining workflows and minimizing user effort.^[^ ^77^, ^78^ ^]^ Automated image analysis, such as in Figure 4c ^[^ ^79^, ^80^ ^]^ and particularly when enhanced by machine learning,^[^ ^80^, ^81^, ^82^ ^]^ could enable consistent extraction of precise information, even from large and complex datasets, making IL‐SEM more accessible and scalable.

In tandem with automation, the integration of data from multiple detectors offers an opportunity to enrich the interpretation of material transformations. Each detector provides unique insights, such as variations in morphology, composition, or depth information, and combining these datasets more comprehensively could yield a deeper understanding of structural and compositional changes. Efforts to simplify and standardize such integrations could expand the utility of IL‐SEM across diverse applications.

Another critical enhancement involves the design of specialized sample holders that ensure consistent orientation of samples across sessions. Precision alignment markers or mechanical guides could eliminate common errors in positioning, simplifying the relocation process and improving reproducibility. Coupled with this, the incorporation of Python scripts into SEM control systems could enable customized workflows, automation of repetitive tasks, and seamless integration with advanced data analysis tools, further enhancing efficiency and accuracy.

Machine learning also presents transformative potential for IL‐SEM workflows by facilitating feature recognition, image alignment, and data interpretation.^[^ ^80^, ^81^, ^82^, ^83^, ^84^ ^]^ Trained models could identify subtle changes across datasets that may elude manual analysis, offering new insights into material behavior and supporting more nuanced research questions.

Continued advancements in SEM hardware—such as increased resolution, detector sensitivity, and stage control—will further enhance the technique's precision and applicability. These improvements, along with the development of open‐access repositories for IL‐SEM datasets, could promote collaboration, inspire new research directions, and provide valuable resources for AI training and validation.

To fully realize IL‐SEM's potential, its adoption must extend across disciplines. By integrating IL‐SEM into more research workflows and fostering a culture of shared knowledge and collaboration, the field can accelerate its development and broaden its impact. Addressing these perspectives will enable IL‐SEM to become an even more powerful and widely utilized tool, unlocking transformative insights into material transformations and driving innovation in fields like catalysis, energy storage, and beyond.

## 3 Conclusion

IL‐SEM enables the direct and reliable observation of morphological and structural transformations at the same nanoscale region. By observing the exact same area before and after, IL‐SEM eliminates spatial uncertainty due to material inhomogeneities. In this work, we present a streamlined and accessible IL‐SEM protocol, supported by detailed practical guidance on orientation strategies, stage rotation, location selection, multi‐scale imaging, and consistent documentation through maps, photos, and coordinate tracking. When forward thinking is applied at every stage—from initial imaging to sample treatment and reanalysis—IL‐SEM becomes a fast and straightforward approach that integrates easily into standard SEM workflows, requiring minimal additional time while offering significant analytical precision. To demonstrate IL‐SEM's applicability and capability, we applied it to analyze a wide range of morphological and structural transformations at the same nanoscale region. A series of advanced and challenging examples positions IL‐SEM as pivotal tool in identifying and confirming the nature of transformations: i) phase‐specific evolution in multicomponent alloys, ii) the first IL‐SEM investigation on gas diffusion electrodes with Pt, iii) automated image‐based correlation of catalyst restructuring with CO_2_ reduction selectivity, iv) reliable compositional tracking throughout synthesis using IL‐EDS analysis and mapping, and, most notably, v) we present the first integration of IL‐SEM with IL‐EBSD to resolve grain‐ and defect‐specific transformations in microstructured materials, positioning this correlative approach as a powerful technique for surface‐sensitive, site‐specific characterization. These contributions, together with the practical and transferable workflow established in this study, firmly position IL‐SEM as a high‐precision, non‐destructive, and straightforward method for uncovering dynamic material transformations across a broad range of advanced research fields.

## 4 Experimental Section

The FeCoNiMoCu (Figure 4a) alloy sample was prepared by standard metallurgical procedures. The arc melting of the pure elements was carried out in a Ti‐getter Ar atmosphere. The ingots were heated five times above the liquidus temperature and cut into 3 mm‐thick and 5 mm‐diameter cylinders. The surface was polished through sequential grinding with SiC papers (320, 800, 1200 grit) and diamond suspensions (9, 3, and 1 µm), followed by final polishing with colloidal silica (∼0.04 µm) to achieve a deformation‐free surface. The sample was then nitridated at 650 °C for 30 min under a continuous flow of ammonia (100 cm^3^ min^−1^) with controlled heating and cooling rates (10 °C min^−1^).

Pt nanoparticles on GDL (Figures 3a and 4b) were prepared by spray coating a Pt‐based ink onto a GDL (Sigracet 39BB, FuelCellStore). The ink was prepared by dispersing 14.75 mg Pt/C catalyst (50% Pt/C, Johnson Matthey Technology Center) into a solution consisting of 7.38 mL isopropanol, 7.38 mL miliQ, and 111.7 µL of ionomer (5% wt D520 Nafion Solution, Ion Power) by 15‐min sonication. The measurements were conducted with a potentiostat (CS310X, CorrTest) in a gas diffusion electrode setup. The geometric area was 0.0707 cm^2^ (3 mm diameter circle). The counter electrode was a titanium mesh coated with mixed metal oxides Ir/Ta (Metakem), and as a reference electrode, RHE (Hydroflex, Gaskatel) was used. The measurements were performed in 2 M HClO_4_ (Supra qualität, ROTH) at room temperature. The catalyst was subjected to a break‐in protocol of 50 cyclovoltammetry cycles between 0.05 – 1.2 V versus RHE at 200 mVs^−1^ in Ar. Then, the sample was subjected to 10 000 AST cycles at 0.95 and 0.55 V for 3 s each.

Cu nanoparticles on a carbon support (Figures 1 and 4c, Figures S1, and S2, Supporting Information) were prepared following the protocol described in ref.^[^ ^11^ ^]^ The electrochemical cell and protocol were identical to those used in the cited work. Electrolysis was conducted for 1 h at the following applied potentials: −0.93 V versus RHE in Figure 1, −0.895 V versus RHE in Figure 4c, −0.975 V versus RHE in Figures S1 and S2 (Supporting Information).

The FeCoNiCuZn high‐entropy alloy catalyst (Figure 5a and Figure S3, Supporting Information) was prepared by pulse electrodeposition onto a Fe substrate (Baoji Lyne Metals Co., Ltd, China) from an acidic sulfate‐based electrolyte (pH ∼3) containing metal sulfates (FeSO_4_ from Alfa Aesar, CoSO_4_ and NiSO_4_ from Sigma–Aldrich, CuSO_4_ from Merck, and ZnSO_4_ from Sigma–Aldrich), with additional chloride for Ni (Merck) and additives Na_3_C_6_H_5_O_7_ (Sigma–Aldrich), C_6_H_8_O_7_ (Sigma–Aldrich), and H_3_BO_3_ (Sigma–Aldrich). Deposition was performed at 1.5 V with 50 ms on‐time and 10 ms off‐time for 18 h, using a Ni cathode (Baoji Lyne Metals Co., Ltd, China) and an Ag/AgCl reference electrode (Corrtest, China). The deposited films underwent nitridation at 700 °C for 30 min under a continuous ammonia flow (100 cm^3^ min^−1^) at atmospheric pressure, using heating/cooling rates of 10 °C min^−1^. Anodization was carried out at 50 V for 5 min in a custom electrochemical cell, following the procedure described by Suhadolnik et al.,^[^ ^85^ ^]^ with samples mounted in a Teflon holder and exposed to freshly prepared electrolyte. Electrochemical testing was performed at room temperature in a single‐compartment cell with a three‐electrode configuration. The sample, mounted in the same custom‐made Teflon holder used for anodization, served as the working electrode. An RHE (Hydroflex, Gaskatel) and a glassy carbon rod were used as the reference and counter electrodes, respectively. The potential was controlled with a Biologic SP‐300 potentiostat. The electrolyte was 1 M KOH (Suprapur, Merck). The activation procedure consisted of 15 cyclic voltammetry cycles between 0.1 and 1.2 V versus RHE at a scan rate of 20 mV s^−1^. Subsequently, OER‐relevant cycling was performed in three stages: initially 2 CV cycles between 1.2 and 1.55 V at a scan rate of 2 mV s^−1^, followed by 150 CV cycles at 50 mV s^−1^ within the same potential window, and finally another 2 CV cycles at 2 mV s^−1^. Electrolyte resistance was measured by electrochemical impedance spectroscopy, and 85% iR compensation was applied.

Inconel 625 (ArcelorMittal) (Figure 5b) was prepared first by cutting out the flat disc‐shaped samples with a diameter of 5 mm and a height of 3 mm. The specimen was sequentially ground with SiC abrasive papers (#320 to #1200) under running water to prevent overheating, followed by polishing with diamond suspensions of 9, 3, and 1 µm on appropriate cloths. Final polishing was performed with colloidal silica suspension (OP‐S, ∼0.04 µm) for 10–20 min to achieve a deformation‐free, highly reflective surface suitable for SEM and EBSD analysis. After each step, samples were rinsed with ethanol and dried with compressed air to prevent cross‐contamination. IL‐SEM, IL‐EBSD, and IL‐EDS mapping analyses were performed before and after extended electrochemical cycling (50 cycles between 0.1 and 1.2 V vs. RHE, followed by 300 cycles in the 1.2–1.55 V vs. RHE range). In a separate preparation route (Figure 3b), the alloy was only ground with SiC paper (#320) before being nitridated at 750 °C for 30 min under a continuous flow of ammonia (100 cm^3^ min^−1^), with controlled heating and cooling rates of 10 °C min^−1^.

All samples were rinsed, dried, and stored under controlled conditions to prevent contamination or degradation before, during, and after analysis.

### IL‐SEM Characterization

IL‐SEM imaging was performed using a ThermoFisher Apreo 2S microscope (Thermo Fisher Scientific, The Netherlands) and a FE‐SEM Supra 35 VP (Zeiss, Germany), both equipped with an EDS detector Ultim Max 100 (Oxford, UK). Surface and morphology images were acquired using different detectors provided for each image, together with the magnifications and accelerating voltages in Tables S1–S5 in the Supporting Information. A Zeiss CrossBeam 550 field‐emission scanning electron microscope (FIB‐SEM, Carl Zeiss, Oberkochen, Germany) equipped with an EDAX Hikari Super EBSD camera was used for the EBSD analysis. An accelerating voltage of 15 kV was applied, with a probe current of 10 nA to obtain detailed crystallographic properties and phase distributions.

The general IL‐SEM workflow included the following steps:

1.1. Sample insertion and alignment: The sample was inserted into the SEM chamber, and stage alignment was achieved using distinct surface features, such as polishing lines or edge indentations, for reliable orientation.

1.2. Initial imaging: Images were captured at the selected nano‐location, with magnifications ranging from 200 000 × to 40 ×, using four detectors (T1, T2, T3, and ETD), corresponding to a scaling factor relative to a 5″ Polaroid reference.

2. Sample retraction for processing: The sample was removed from the SEM chamber for treatments such as heat treatment or electrochemical testing.

3. Re‐identification of the identical location: The previously recorded imaging dataset served as a reference to gradually re‐locate the identical area, narrowing down from macro to nano scales.

4. Post‐treatment imaging and analysis: The identical location was re‐imaged at the same set of magnifications and detector configurations used in the initial session to ensure consistent and comparable results.

EDS analysis was conducted at the identical location using a selected magnification and voltage. In certain cases, large area EDS mapping was performed on the same region, covering a larger area by analyzing multiple smaller regions and combining the data into a single comprehensive image. This mapping process often required an overnight operation to acquire high‐resolution data.

### Data Management

Images, analytical data, and accompanying notes—including observations on results, descriptions of identical location identification, and details on sample processing—were systematically recorded and stored using the Qx application (Quipnex, Ljubljana, Slovenia). This ensured structured data organization, facilitating efficient tracking of sample history and enabling seamless comparison between different IL‐SEM sessions.

## Conflict of Interest

The authors declare no conflict of interest.

## Supporting information



Supporting Information

## Data Availability

The data that support the findings of this study are available from the corresponding author upon reasonable request.
