# Supplementary material for: Workflow and Practical Guidance for Identical Location Scanning Electron Microscopy: Reliable Tracking of Localized Transformations
Source: Small Methods. 2025 Aug 13;9(9):e01290. doi: 10.1002/smtd.202501290 (PMC12464639; doi:10.1002/smtd.202501290)
Supplement: Supplementary file 1 — Supporting Information [file SMTD-9-e01290-s001.docx]

Supporting Information

Workflow and Practical Guidance for Identical Location Scanning Electron Microscopy: Reliable Tracking of Localized Transformations

Blaž Tomc, Marjan Bele*, Ana Rebeka Kamšek, Milena Martins, Aleš Marsel, Miha Hotko, Gregor Kapun, Stefan Popović, Črtomir Donik, Mitja Kostelec, Matjaž Godec, Nejc Hodnik*, Luka Suhadolnik*


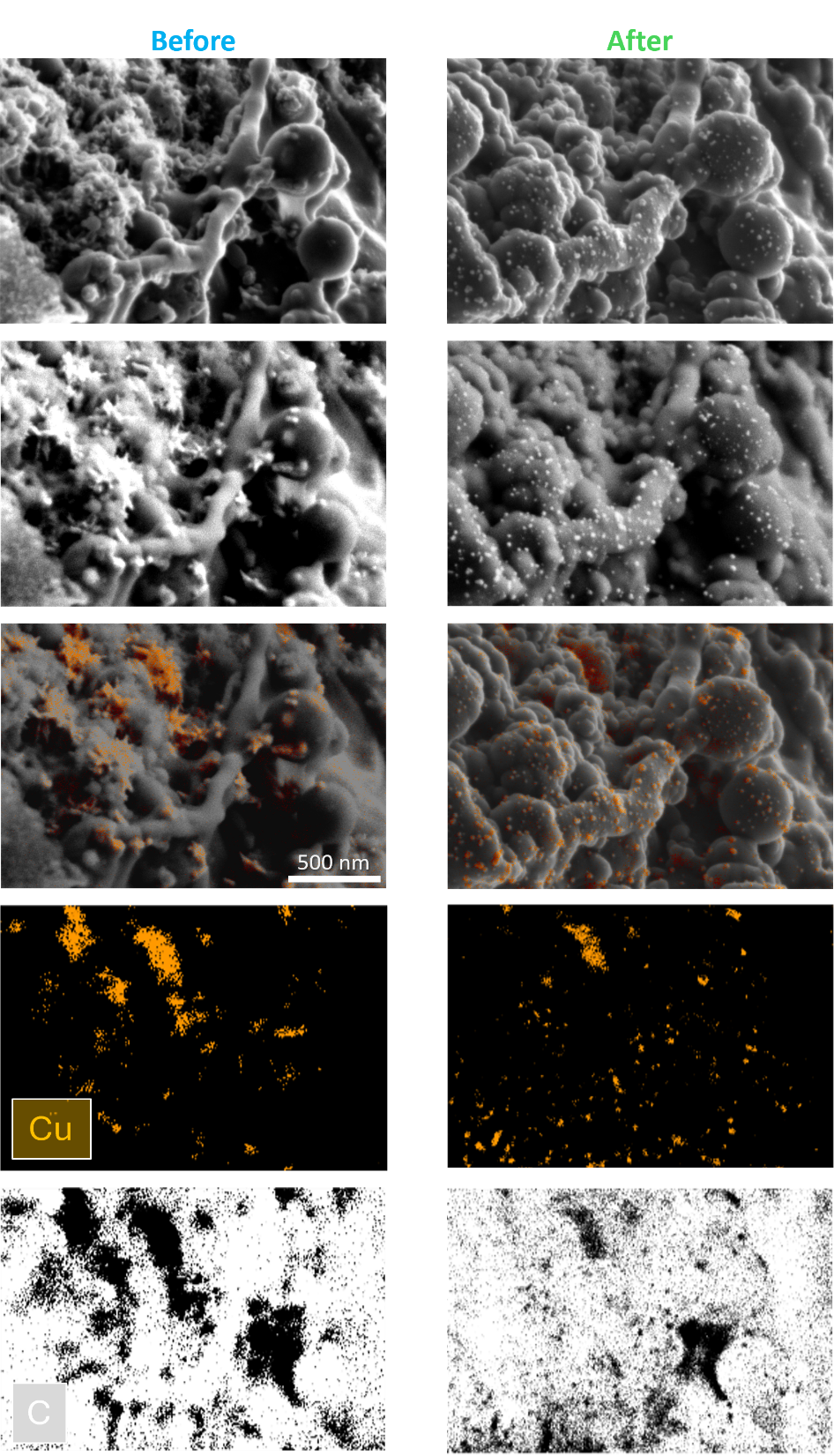


Figure S1: Copper nanoparticles before and after CO_2_RR with noticeable SEM electron beam-induced carbon deposition after 2 hours of EDS mapping.


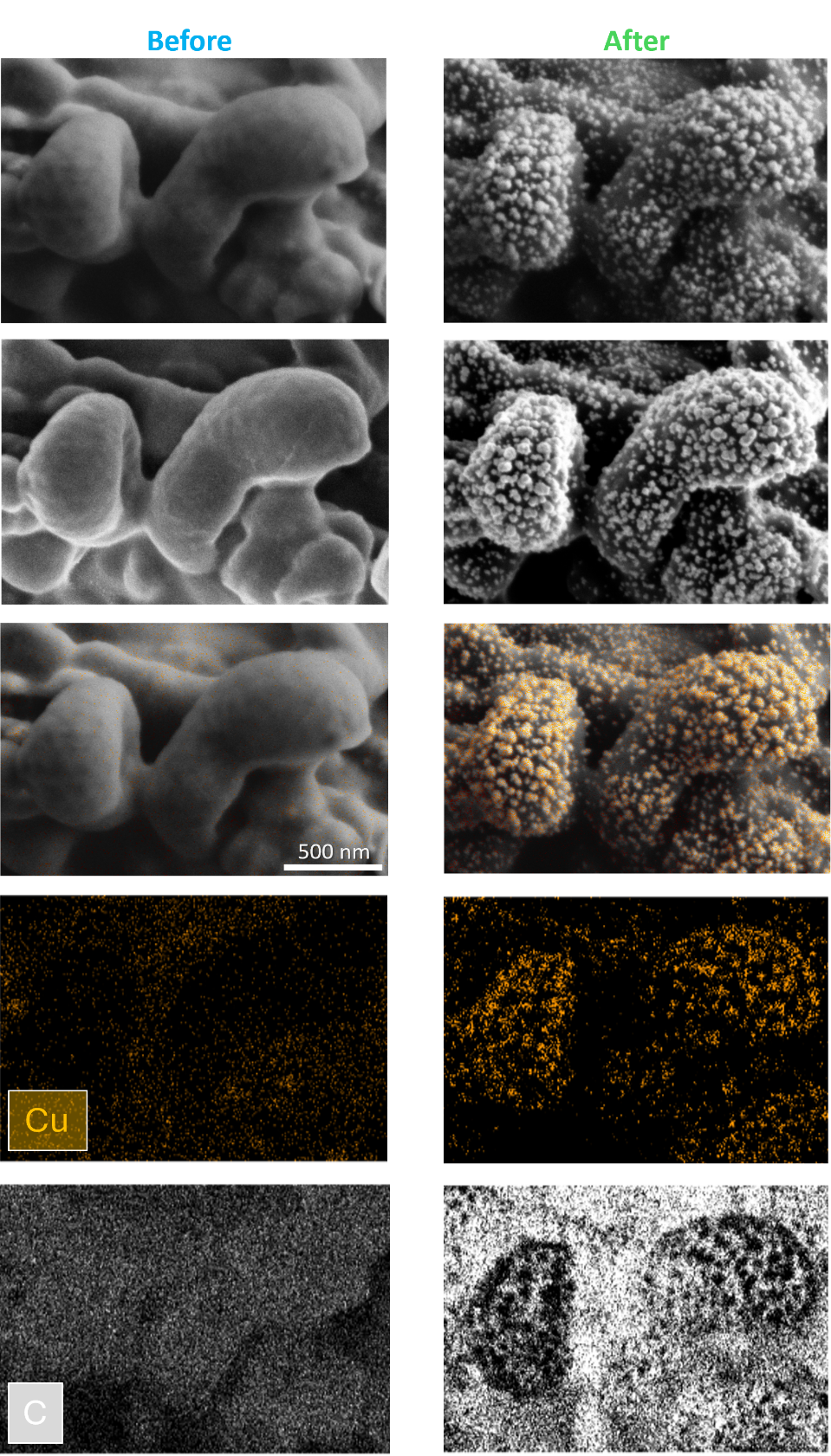


Figure S2: Copper nanoparticles before and after CO_2_RR, without any noticeable SEM electron beam-induced carbon deposition, when EDS mapping was performed for 10 minutes.


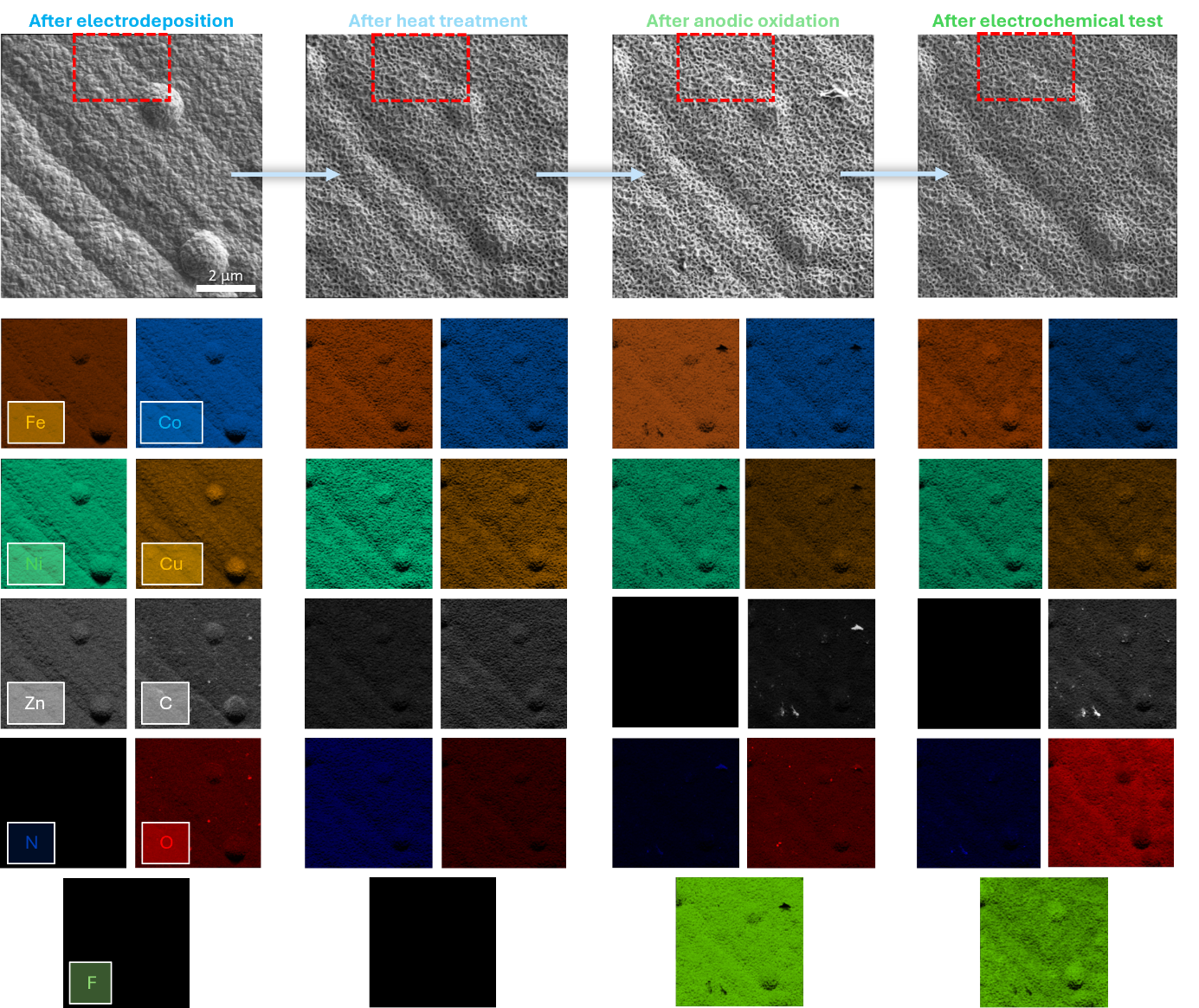


Figure S3: IL-SEM-EDS mapping of the same location after various treatments of bulk FeCoNiCuZn OER alloy.


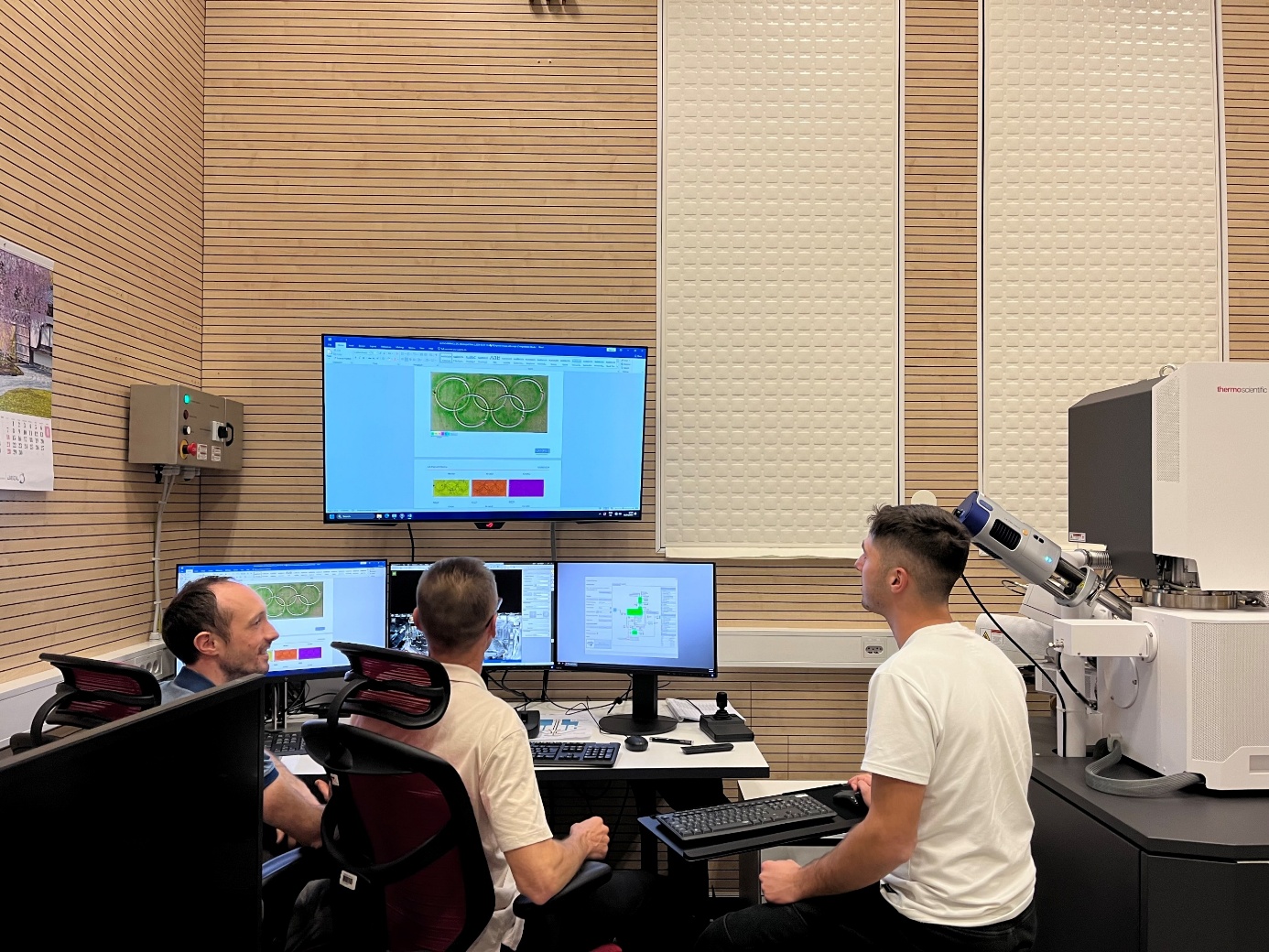


Figure S4: Collaborative IL-SEM analysis involving a “driver” and “navigators.” This teamwork approach enhances efficiency, ensuring precise identification of identical locations while fostering real-time discussion and idea generation during SEM imaging.

Table S1: Microscope model, magnifications, accelerating voltages, detector settings, and imaging conditions used for surface and morphology imaging of samples shown in Figure 2 in the main text.

| Figure 2 | | | | |
| --- | --- | --- | --- | --- |
| Mark | Voltage [kV] | Magnification | Detector | SEM |
| 1.1 | 7 | 200k | InLens | FE-SEM Supra 35 VP |
| 1.2.1 | 7 | 50k | InLens | FE-SEM Supra 35 VP |
| 1.2.2 | 7 | 10k | InLens | FE-SEM Supra 35 VP |
| 1.2.3 | 7 | 2k | SE2 | FE-SEM Supra 35 VP |
| 1.2.4 | 7 | 400 | SE2 | FE-SEM Supra 35 VP |
| 1.2.5 | 7 | 100 | SE2 | FE-SEM Supra 35 VP |
| 3.1 | 7 | 80 | SE2 | FE-SEM Supra 35 VP |
| 3.2 | 7 | 500 | SE2 | FE-SEM Supra 35 VP |
| 3.3 | 7 | 2K | SE2 | FE-SEM Supra 35 VP |
| 3.4 | 7 | 10K | SE2 | FE-SEM Supra 35 VP |
| 3.5 | 7 | 50K | InLens | FE-SEM Supra 35 VP |
| 3.6 | 7 | 200k | InLens | FE-SEM Supra 35 VP |
| 4.1 | 7 | 300K | InLens | FE-SEM Supra 35 VP |
| 4.2 | 7 | 300K | InLens | FE-SEM Supra 35 VP |
| 4.3 | 7 | 50K | SE2 | FE-SEM Supra 35 VP |
| 4.4 | 7 | 50k | SE2 | FE-SEM Supra 35 VP |

Table S2: Microscope model, magnifications, accelerating voltages, detector settings, and imaging conditions used for surface and morphology imaging of samples shown in Figure 3 in the main text.

| Figure 3 | | | | |
| --- | --- | --- | --- | --- |
| Mark | Voltage [kV] | Magnification | Detector | SEM |
| b | 5 | 40 | ETD | ThermoFisher Apreo 2S |
| c | 5 | 100 | T1 | ThermoFisher Apreo 2S |
| d | 7 | 77 | InLens | ThermoFisher Apreo 2S |
| e | 5 | 100 | ETD | ThermoFisher Apreo 2S |
| f | 5 | 100 | ETD | ThermoFisher Apreo 2S |
| h | 2 | 47 | T1 | ThermoFisher Apreo 2S |
| i | 3 | 218 | InLens | FE-SEM Supra 35 VP |

Table S3: Microscope model, magnifications, accelerating voltages, detector settings, and imaging conditions used for surface and morphology imaging of samples shown in Figure 4 in the main text.

| Figure 4 | | | | |
| --- | --- | --- | --- | --- |
| Mark | Voltage [kV] | Magnification | Detector | SEM |
| a1 | 5 | 100k | ETD | ThermoFisher Apreo 2S |
| a2 | 5 | 100k | ETD | ThermoFisher Apreo 2S |
| a3 | 5 | 100K | T1 | ThermoFisher Apreo 2S |
| a4 | 5 | 100k | T1 | ThermoFisher Apreo 2S |
| a5 | 5 | 100k | T2 | ThermoFisher Apreo 2S |
| a6 | 5 | 100k | T2 | ThermoFisher Apreo 2S |
| a7 | 5 | 100k | T3 | ThermoFisher Apreo 2S |
| a8 | 5 | 100k | T3 | ThermoFisher Apreo 2S |
| b1 | 5 | 2k | ETD | ThermoFisher Apreo 2S |
| b2 | 5 | 2k | ETD | ThermoFisher Apreo 2S |
| b3 | 5 | 2k | T1 | ThermoFisher Apreo 2S |
| b4 | 5 | 2k | T1 | ThermoFisher Apreo 2S |
| b5 | 5 | 2k | T2 | ThermoFisher Apreo 2S |
| b6 | 5 | 2k | T2 | ThermoFisher Apreo 2S |
| b7 | 5 | 2k | T3 | ThermoFisher Apreo 2S |
| b8 | 5 | 2k | T3 | ThermoFisher Apreo 2S |

Table S4: Microscope model, magnifications, accelerating voltages, detector settings, and imaging conditions used for surface and morphology imaging of samples shown in Figure 5 in the main text.

| Figure 5 | | | | |
| --- | --- | --- | --- | --- |
| Mark | Voltage [kV] | Magnification | Detector | SEM |
| a1 | 5 | 2k | ETD | ThermoFisher Apreo 2S |
| a2 | 5 | 2k | ETD | ThermoFisher Apreo 2S |
| b1 | 5 | 200k | T3 | ThermoFisher Apreo 2S |
| b2 | 5 | 200k | T3 | ThermoFisher Apreo 2S |
| c1 | 7 | 100k | InLens | FE-SEM Supra 35 VP |
| c2 | 7 | 100k | InLens | FE-SEM Supra 35 VP |

Table S5: Microscope model, magnifications, accelerating voltages, detector settings, and imaging conditions used for surface and morphology imaging of samples shown in Figure 6 in the main text.

| Figure 6 | | | | |
| --- | --- | --- | --- | --- |
| Mark | Voltage [kV] | Magnification | Detector | SEM |
| a1 | 5 | 2k | ETD | ThermoFisher Apreo 2S |
| a2 | 5 | 2k | ETD | ThermoFisher Apreo 2S |
| a3 | 5 | 2k | ETD | ThermoFisher Apreo 2S |
| a4 | 5 | 2k | ETD | ThermoFisher Apreo 2S |
| b1 | 15 | 1k | SE2 | Zeiss CrossBeam 550 |
| b2 | 15 | 1k | BSD1 | Zeiss CrossBeam 550 |

Table S6: Magnifications and accelerating voltages used for EDS analysis of samples shown in this paper.

| Figure | Voltage [kV] | Individual image magnification | Sites shown in figure | Montage magnification | Total number of sites |
| --- | --- | --- | --- | --- | --- |
| 6a | 5 | 8k | 1 | 1.5k | 49 |
| 6b | 5 | 7k | 18 | 1k | 81 |
| S1 | 2 | 65k | 1 | 65k | 1 |
| S2 | 2 | 65k | 1 | 65k | 1 |
| S3 | 5 | 8k | 20 | 1.5k | 49 |
